# Global Variations in Event-Based Surveillance for Disease Outbreak Detection: Time Series Analysis

**DOI:** 10.2196/36211

**Published:** 2022-10-31

**Authors:** Iris Ganser, Rodolphe Thiébaut, David L Buckeridge

**Affiliations:** 1 McGill Clinical and Health Informatics School of Population and Global Health McGill University Montreal, QC Canada; 2 INSERM U1219 Bordeaux Population Health Research Center INRIA 2 Université de Bordeaux Bordeaux France; 3 Service d’Information Médicale Centre Hospitalier Universitaire de Bordeaux Bordeaux France

**Keywords:** event-based surveillance, digital disease detection, public health surveillance, influenza, infectious disease outbreak, surveillance, disease, outbreak, analysis, public health, data, detection, detect, epidemic

## Abstract

**Background:**

Robust and flexible infectious disease surveillance is crucial for public health. Event-based surveillance (EBS) was developed to allow timely detection of infectious disease outbreaks by using mostly web-based data. Despite its widespread use, EBS has not been evaluated systematically on a global scale in terms of outbreak detection performance.

**Objective:**

The aim of this study was to assess the variation in the timing and frequency of EBS reports compared to true outbreaks and to identify the determinants of variability by using the example of seasonal influenza epidemic in 24 countries.

**Methods:**

We obtained influenza-related reports between January 2013 and December 2019 from 2 EBS systems, that is, HealthMap and the World Health Organization Epidemic Intelligence from Open Sources (EIOS), and weekly virological influenza counts for the same period from FluNet as the gold standard. Influenza epidemic periods were detected based on report frequency by using Bayesian change point analysis. Timely sensitivity, that is, outbreak detection within the first 2 weeks before or after an outbreak onset was calculated along with sensitivity, specificity, positive predictive value, and timeliness of detection. Linear regressions were performed to assess the influence of country-specific factors on EBS performance.

**Results:**

Overall, while monitoring the frequency of EBS reports over 7 years in 24 countries, we detected 175 out of 238 outbreaks (73.5%) but only 22 out of 238 (9.2%) within 2 weeks before or after an outbreak onset; in the best case, while monitoring the frequency of health-related reports, we identified 2 out of 6 outbreaks (33%) within 2 weeks of onset. The positive predictive value varied between 9% and 100% for HealthMap and from 0 to 100% for EIOS, and timeliness of detection ranged from 13% to 94% for HealthMap and from 0% to 92% for EIOS, whereas system specificity was generally high (59%-100%). The number of EBS reports available within a country, the human development index, and the country’s geographical location partially explained the high variability in system performance across countries.

**Conclusions:**

We documented the global variation of EBS performance and demonstrated that monitoring the report frequency alone in EBS may be insufficient for the timely detection of outbreaks. In particular, in low- and middle-income countries, low data quality and report frequency impair the sensitivity and timeliness of disease surveillance through EBS. Therefore, advances in the development and evaluation and EBS are needed, particularly in low-resource settings.

## Introduction

Infectious diseases continue to threaten populations worldwide, as demonstrated clearly during the novel coronavirus (SARS-CoV-2) pandemic. Infectious disease surveillance produces crucial information for public health professionals to make good emergency response decisions and implement effective countermeasures to contain outbreaks [1,2]. Traditional disease surveillance (or indicator-based surveillance [IBS]) relies on laboratory test results transmitted through public health networks [3,4], but these systems are focused on only a few diseases, can have a considerable reporting time lag, and lack sensitivity, especially for novel pathogens [5-7]. Event-based surveillance (EBS) was developed to complement IBS to enable near real-time detection of infectious disease outbreaks [3]. To identify possible outbreaks, EBS attempts to detect unusual patterns related to potential events, which precede the official confirmation of disease outbreaks [2,8]. For this purpose, EBS systems use unstructured and mainly internet-based data such as web-based news articles [9,10].

In addition to early disease activity detection, surveillance for events has the potential to augment the sensitivity of IBS in regions with few medical centers or lower health-seeking behaviors [11]. The usefulness of EBS in resource-limited settings has been demonstrated by its ability to detect dengue fever [12] and Ebola outbreaks [13,14] before detection by official bodies. Furthermore, resource-limited regions stand to benefit the most from EBS, as they are disproportionately affected by infectious diseases [15] and may have limited resources to implement IBS systems. However, due to the unstructured and unverified nature of the gathered data, EBS systems face considerable challenges, with the overarching problem being the accurate discrimination of true signals from an immense amount of noise [16,17]. EBS systems are also highly dependent on the internet coverage in the countries of operation and filtering of languages. This may lead to a considerable variation in EBS performance across geographical settings, which, together with the inherent uncertainty of the information gathered, suggests that EBS systems should be carefully evaluated in a representative set of countries. However, despite their widespread use, EBS systems tend to be used in an ad hoc and informal way. This type of use could explain why there is not much published evidence about the variation in EBS system performance across countries. Rather, most available literature focus on the adequate classification of health-related events from web-based sources or the implementation of innovative functionalities [18-20].

Therefore, as the first objective, we aimed to document the global variation in the sensitivity and timing of the information obtained through EBS for outbreak detection. We applied a systematic monitoring approach to data derived from 2 EBS systems in a representative set of countries and used seasonal influenza outbreaks as the test case. As our second objective, we identified factors driving the observed differences in detection across countries. The identification of factors that influence performance is important to identify ways to improve EBS, especially in resource-poor settings.

## Methods

### Data

For this study, 24 countries from 15 influenza transmission zones were chosen to evaluate EBS performance on a global scale: Argentina, Australia, Brazil, Bulgaria, China, Costa Rica, Ecuador, Egypt, France, Germany, Greece, India, Iran, Mexico, Nigeria, Russia, Saudi Arabia, South Africa, Sweden, Thailand, Uruguay, United Kingdom, United States, and Vietnam. These countries were selected to represent a broad spectrum of geographical locations, languages, and income brackets and were sampled randomly from all influenza transmission zones.

### FluNet: The Reference

FluNet is a web-based tool created by the World Health Organization (WHO) for disseminating virologic influenza surveillance data and serves as the reference to evaluate EBS systems. FluNet provides publicly available counts of laboratory-confirmed influenza cases per country, aggregated per week, from all participating global influenza surveillance and response system (GISRS) countries, other national influenza reference laboratories, which are collaborating with GISRS, and from WHO regional databases [21]. FluNet data were collected from January 2013 to December 2019, except for Saudi Arabia, where FluNet data were only available as of January 2017. The beginning of the study period was chosen to be January 2013 so that the effects of the 2009 pandemic would not influence the analysis.

### Ethical Considerations

As FluNet is an active international surveillance tool and its data are published in an aggregated manner in the open domain, the approval of a research ethics board was not required for this research.

### HealthMap Data

HealthMap provides real-time surveillance of infectious diseases by collecting data from web-based news aggregators such as Google News, expert-moderated systems such as Program for Monitoring Emerging Diseases-Mail, and validated alerts from official sources [10,19]. Any news article that passed through HealthMap’s filtering algorithm from January 2013 to July 2019 and related to “human influenza” was considered an influenza report. Duplicate reports were identified by a unique ID number assigned to each report and removed together with reports concerning countries’ overseas territories (9012 and 62 out of 31,796 total events, respectively), resulting in a total of 22,722 unique reports. To match gold standard data, daily report counts from HealthMap were aggregated into a weekly format, resulting in a total of 341 weekly data points spanning 6.5 years.

### Epidemic Intelligence From Open Sources Data

In 2017, the WHO implemented the Epidemic Intelligence from Open Sources (EIOS) system as a collaboration between multiple public health organizations, acting on a global scale to provide timely public health surveillance [22]. EIOS integrates data from multiple EBS systems, including HealthMap, and performs report deduplication before uploading to the platform. Daily event data were provided from November 11, 2017 (the day of EIOS implementation) through December 2019. All EIOS reports for the 24 countries of interest with the keywords “Influenza virus not identified,” “H1N1,” “H1N1v,” “H1N2,” “H1N2v,” “H2N1,” “H2N2,” “H3N2,” and “H3N2v” were retrieved and aggregated into weeks, totaling 109 weeks.

### Outbreak Detection Methodology and Workflow

**Figure 1:**
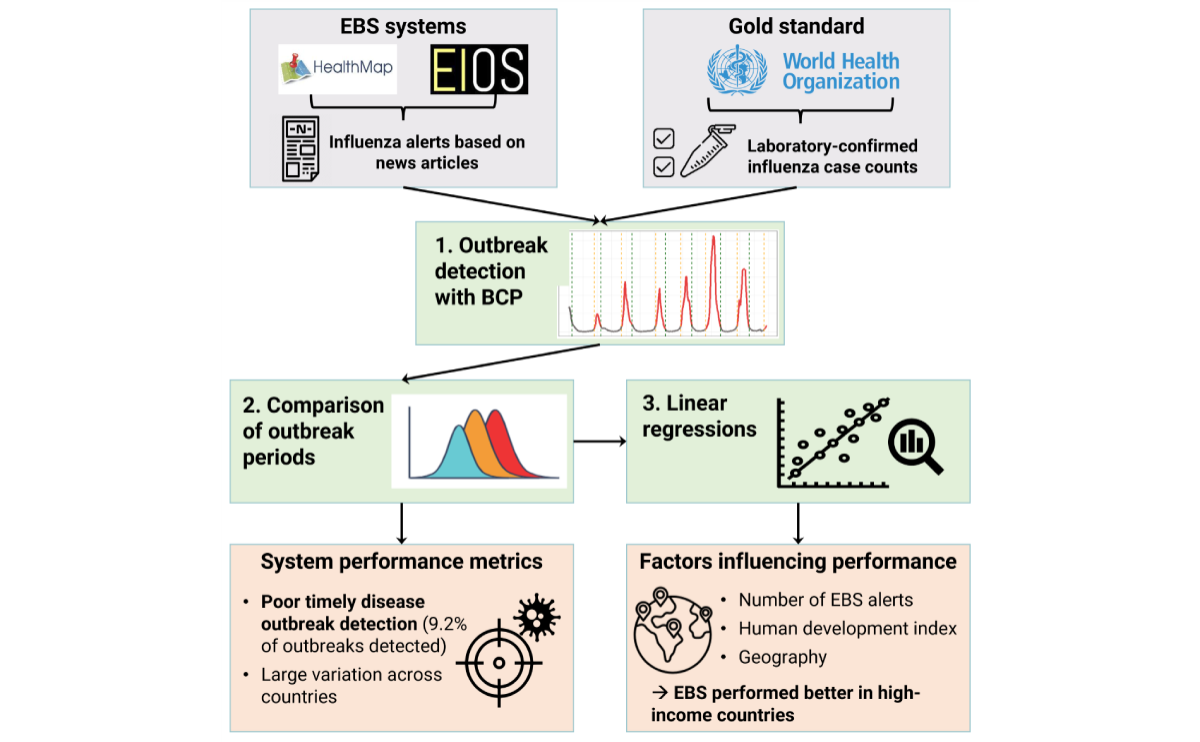
Illustration of the workflow. BCP: Bayesian change point analysis; EBS: event-based surveillance.

Our overall workflow is illustrated in Figure 1. Outbreaks were detected retrospectively in all 3 data sets based on media report frequency or confirmed influenza case counts by using Bayesian change point (BCP) analysis. The date of outbreak onset had to be detected in gold standard as well as in EBS data because FluNet did not provide a consistent epidemic indicator. Although not initially developed for infectious disease outbreaks [23,24], change point analysis has been used to determine the start points of influenza epidemics [25,26]. Essentially, change point methods identify points in time series before and after which distributional parameters differ significantly. To do this, the BCP algorithm breaks a time series into blocks, calculates the mean and variance of these blocks, and derives the probability of each break point being a change point. All BCP analyses were conducted using the R package bcp, version 4.0.3 [23,27], which is based on the method described by Barry and Hartigan [24]. The posterior mean and variance, and from this, the probability of each time point being a change point, were estimated with 600 Markov Chain Monte Carlo iterations, and the first 100 Markov Chain Monte Carlo iterations were discarded as burn-in. Only 600 iterations were used, as convergence was reached quickly after the first iterations. The priors p0 (change point probability) and w0 (signal-to-noise ratio) were kept at their default value of 0.2. As multiple change points were flagged by BCP analysis during outbreaks, we applied additional criteria to determine the start and end points of influenza outbreaks. In short, epidemic start and end points were the first and last change points in the rising and descending curves, respectively. See Multimedia Appendix 1 for a more detailed description of the BCP method and our outbreak detection algorithm.

### Performance Evaluation Metrics

The epidemic intervals detected using HealthMap and EIOS reports were compared to the intervals detected using FluNet to calculate sensitivity (the proportion of all outbreaks in the gold standard data detected by the EBS systems), positive predictive value (PPV, the proportion of all outbreaks detected in the EBS data corresponding to an outbreak in the gold standard), specificity (the proportion of all weeks without an outbreak in the EBS data, during which no outbreak was detected in the gold standard data), timeliness, and timely sensitivity for each EBS system separately by country. The metric of timely sensitivity describes the ability of a system to detect an outbreak before or around the same time as traditional surveillance systems and is defined as outbreak detection by EBS within a window of 2 weeks before and after the start of an outbreak in the gold standard. Our rationale for choosing this time interval around the outbreak onset in the gold standard data is that the reporting lag of IBS data for influenza usually exceeds 2 weeks; so, this is the timeliness advantage possibly gained by EBS. Moreover, we hypothesized that a true outbreak could be detected 2 weeks before outbreak onset in the gold standard data, but not earlier than that, to avoid detection of false positives. Timeliness was defined as the proportion of outbreak duration remaining at detection to circumvent the problem of nondetected outbreaks [28].




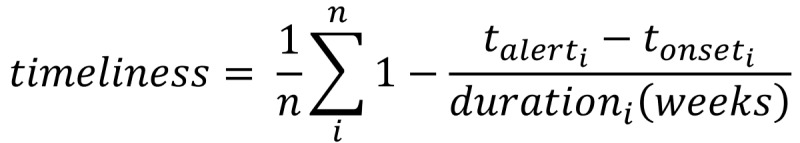




In the equation above, alert refers to a detected outbreak in the EBS system data. n (weeks) is the total number of weeks in the study for the respective system (341 for HealthMap and 109 for EIOS). Accuracy was calculated as the sum of correctly classified weeks over the number of all weeks.

### Regressions and Variable Selection Process

We conducted multivariable regressions to identify the independent effect of country-specific factors (independent variables) on the performance metrics (dependent variables), while controlling for all other factors included in the model. Highly correlated independent variables were removed from the models until the variance inflation factors were below the conservative threshold of 4 [29]. Influential variables were selected in a forward selection process based on the Akaike information criterion. By checking the residual plots of our final models, we confirmed that all model assumptions were met.

Country-specific variables examined as explanatory variables were the total number of media reports over the data collection period, the maximum counts of media reports per week, global region (temperate Northern hemisphere, temperate Southern hemisphere, or tropical), language (official language English yes/no), latitude, longitude, human development index (HDI), Press Freedom Index (PFI), the total number of internet users, and HealthMap filter language (yes/no). Latitude and longitude were assigned to the centroid of each country. Country languages could only be explored as a binary indicator of English as the official language or not because of sparse strata. Geography was explored as a categorical variable (temperate vs tropical) and as a continuous variable (latitude/longitude). HDI rankings from 2018 and the total number of internet users per country in 2017 were obtained from the United Nations Development Programme [30]. PFI values from 2018 were obtained from *Reporters without Borders* [31]. The PFI ranges from 1 to 100, with lower values indicating higher press freedom. All regression models were fit using the R software version 3.6.3 [32], including the packages MASS version 7.3-51.6 [33] and glmnet version 4.0 [34]. Effect estimates are reported as point estimates and 95% CIs, and *P* values are provided for orientation only. In 2 sensitivity analyses, we selected variables based on least absolute shrinkage and selection operator regressions and excluded the 3 countries with low FluNet data quality (Nigeria, Thailand, and Vietnam).

## Results

### Sample Characteristics

Laboratory-confirmed positive influenza counts over 7 years from FluNet were compared to HealthMap reports over 6.5 years and EIOS reports over 2 years. In most countries, the numbers of influenza-positive specimens provided by FluNet were high enough to allow a good distinction between epidemic and nonepidemic periods. In Nigeria, Thailand, and Vietnam, the signal-to-noise ratio was very high due to a low total number of tested individuals and positive results. The number of EBS signals varied by country, and EIOS collected more annual reports than HealthMap due to its aggregation of sources (Multimedia Appendix 2). Few HealthMap reports were collected in 12 countries (Bulgaria, Costa Rica, Ecuador, Germany, Greece, Iran, Nigeria, Saudi Arabia, South Africa, Sweden, Thailand, and Uruguay), in which HealthMap did not filter for news articles in the respective official languages.

**Figure 2:**
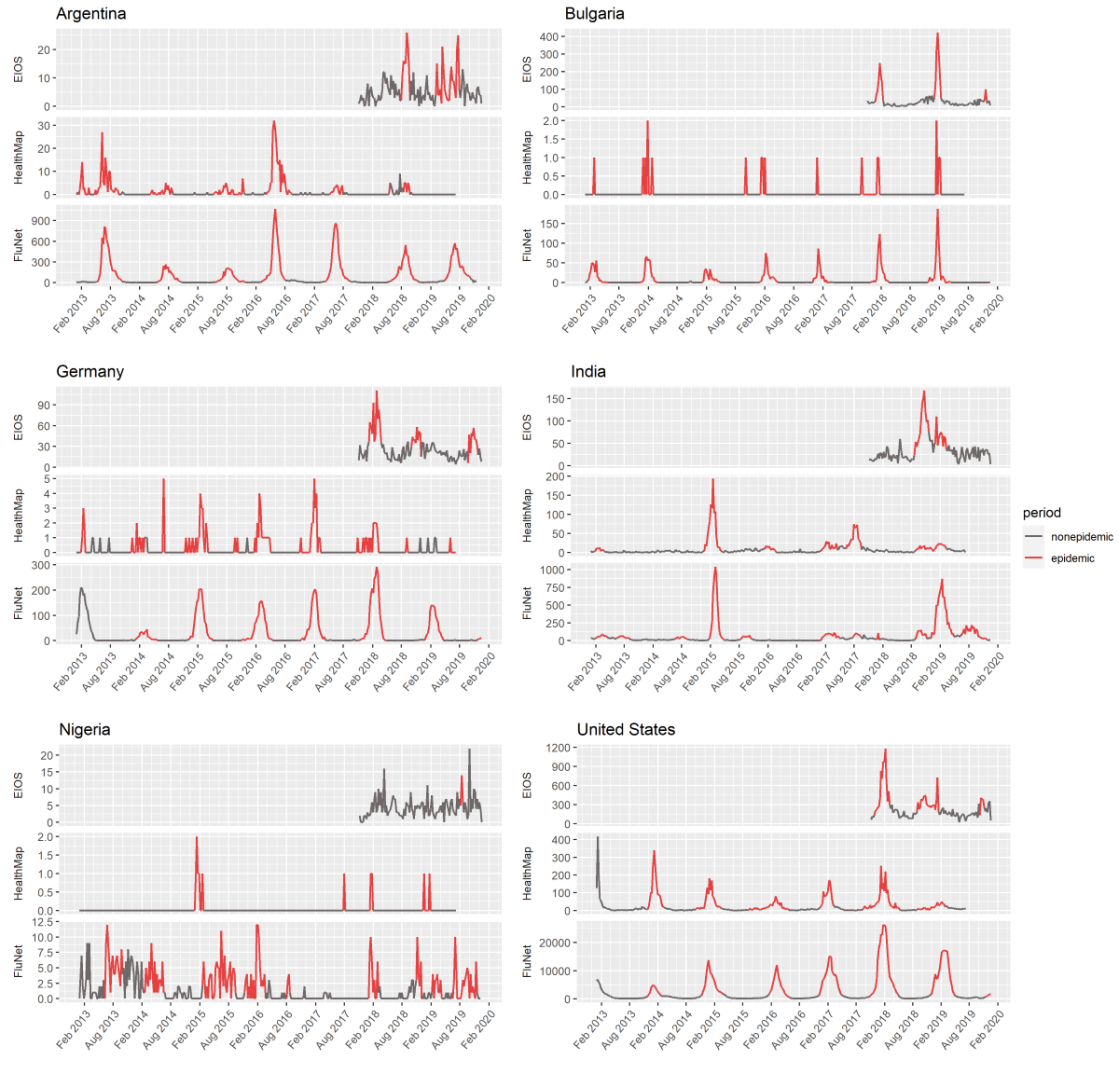
Time series of weekly reports relating to influenza from Epidemic Intelligence from Open Sources and HealthMap and weekly virological influenza counts from FluNet for selected countries from January 2013 to December 2019. Epidemic periods found with Bayesian change point analysis for each system are highlighted in red, and nonepidemic periods are shown in grey. EIOS: Epidemic Intelligence from Open Sources.

Figure 2 illustrates the report frequency over time for FluNet, HealthMap, and EIOS for selected countries. HealthMap reports in countries with frequent reporting generally coincided with influenza epidemics in FluNet data (eg, Argentina, India, United States). In contrast, EIOS reports appeared to be less synchronized with FluNet counts and tended to have a lower signal-to-noise ratio. It is apparent from these plots that there are substantial differences in influenza activity and EBS signals across countries.

### Evaluation of System Performance

**Figure 3:**
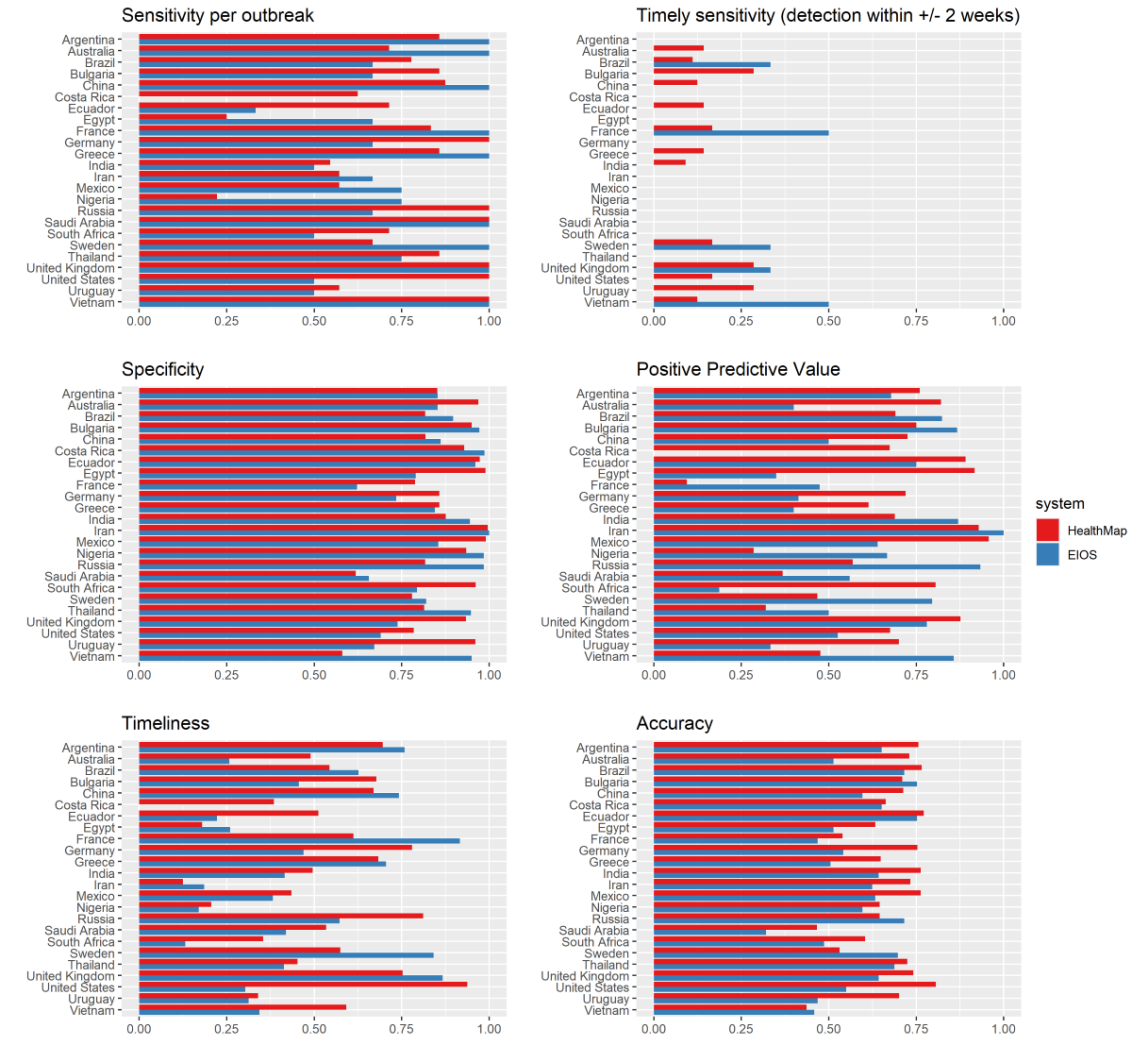
HealthMap and Epidemic Intelligence from Open Sources performance metrics for the detection of influenza outbreaks from January 2013 to July 2019 (HealthMap) or from November 2017 to December 2019 (Epidemic Intelligence from Open Sources). All metrics were calculated with FluNet data as reference. EIOS: Epidemic Intelligence from Open Sources.

Overall, 22 out of 238 (9.2%) outbreaks in the data sets were detected within a time interval of 2 weeks before or after outbreak onset in the gold standard data. HealthMap and EIOS did not detect any outbreaks in a timely manner in 12 (50%) and 19 (79%) out of 24 countries, respectively (Figure 3). HealthMap showed the best timely outbreak detection in Bulgaria, the United Kingdom, and the United States, with 2 out of 7 (29%) detected outbreaks each. EIOS showed a timely sensitivity of 50% in France and Vietnam, corresponding to 1 out of 2 outbreaks detected on time and 33% (1 out of 3 outbreaks detected on time) in Brazil, Sweden, and the United Kingdom. In contrast, the sensitivity of EBS systems was much higher than the timely sensitivity, with 100% of outbreaks detected at any time during the outbreak in 6 countries by HealthMap and 9 countries by EIOS. However, the between-country variation was large. Likewise, PPV and timeliness were very heterogeneous across countries: HealthMap had a PPV <50% in 4 countries and >75% in 9 countries, while EIOS’s PPV ranged from 0% in Costa Rica to 100% in Iran, with 8 countries <50% and 8 countries >75%. Timeliness was especially poor in EIOS, where the proportion of outbreak duration remaining at detection was <50% in 15 countries. This means that most EBS alerts were raised more than halfway through the outbreak, by which point the outbreak would likely be detected through other means.

To gain a more comprehensive understanding of system performance, accuracy was calculated as a metric combining sensitivity and specificity (Figure 3). HealthMap’s accuracy was the highest in the United States (81%), Ecuador, and Brazil (both 77%), and the lowest in Saudi Arabia (47%) and Vietnam (44%). EIOS showed the highest accuracy in Brazil and Russia (both 75%), Bulgaria, and Ecuador (both 72%), and the lowest accuracy in Saudi Arabia (46%) and Vietnam (32%) as well.

As an illustrative example, HealthMap showed consistently high evaluation metrics in the United States because HealthMap reports were well synchronized with seasonal influenza epidemics reflected in FluNet counts (Figure 2 and Multimedia Appendix 3). In a country with no clear influenza seasonality, such as India, HealthMap’s timeliness and sensitivity were lower than that in the United States (timeliness: 50% vs 94%, sensitivity: 55% vs 100%, respectively), but overall accuracy was not markedly reduced (76% vs 81%, respectively) because of high specificity (88% vs 78%, respectively). In contrast, EIOS detected many epidemic signals in the United States in the nonepidemic season of 2018, which decreased its performance metrics relative to India, where EIOS had equal sensitivity (both 50%) but higher specificity (95% vs 69%, respectively). Clearly, a set of country-specific factors must lead to these differences in system performance between the United States and India.

### Identification of Factors Influencing the System Performance

Next, we identified factors associated with the variation of performance indicators across countries stratified by system. It was not possible to identify predictors of timely sensitivity for either system since assumptions of linear regressions were not met and logistic regressions were problematic, as some of the explanatory variables showed perfect separation. Only a moderate amount of the variation in the metrics was explained by the variables examined, as the R^2^ values ranged between 0.144 and 0.587. In HealthMap (Table 1), higher sensitivity was associated with a higher total number of reports and higher HDI. Increasing press freedom (corresponding to a lower PFI) and English as the official language were associated with a lower sensitivity of HealthMap. Higher sensitivity of EIOS (Table 2) was independently associated with higher latitude and lower press freedom. In contrast, a higher HDI reduced HealthMap’s specificity while increasing PFI decreased specificity. EIOS’s specificity was markedly higher in tropical regions than in temperate regions. None of the examined variables was significantly associated with HealthMap’s PPV, while a country’s latitude and PFI influenced EIOS’s PPV. Timeliness of outbreak detection was associated with a higher number of reports, higher HDI, and increasing latitude in HealthMap, and with higher number of reports, increasing latitude, and not having English as the official country language in EIOS. Both sensitivity analyses showed very similar results to those of the main analysis.

**Table 1:** Effect of country-specific covariates on HealthMap performance in detecting influenza outbreaks from January 2013 to July 2019.

Outcome, covariate			Category/increment	Coefficient (95% CI)	*P* value	Adjusted R²
**Sensitivity**						0.456
		Log (total reports)	1 log	0.039 (–0.012 to 0.091)	.13	
		Human development index	1 score	0.017 (0.008 to 0.025)	<.001	
		Press freedom index	1 score	0.003 (–0.001 to 0.008)	.10	
		Official language (reference: not English)	English	–0.128 (–0.313 to 0.057)	.16	
**Positive predictive value**						
	**Specificity**					0.144
		Human development index	1 score	–0.004 (–0.009 to 0.001)	.11	
		Press freedom index	1 score	–0.003 (–0.005 to –0.0003)	.03	
	**Timeliness**					0.504
		Log (total reports)	1 log	0.047 (0.013 to 0.082)	.009	
		Human development index	1 score	0.007 (–0.001 to 0.015)	.08	
		Latitude	1°	0.004 (–0.001 to 0.009)	.12	

**Table 2:** Effect of country-specific covariates on the performance of Epidemic Intelligence from Open Sources in detecting influenza outbreaks from November 2017 to December 2019.

Outcome, covariate		Category/increment	Coefficient (95% CI)	*P* value	Adjusted R²
**Sensitivity**					0.229
	Latitude	1°	0.008 (0.002 to 0.014)	.02	
	Press freedom index	1 score	0.005 (–0.0003 to 0.010)	.06	
**Positive predictive value**					0.587
	Global region (reference: Temperate Northern)	Temperate Southern	–0.008 (–0.219 to 0.203)	.94	
	Global region (reference: Temperate Northern)	Tropical	0.481 (0.271 to 0.692)	<.001	
	Latitude	1°	0.012 (0.006 to 0.018)	<.001	
	Press freedom index	1 score	0.003 (–0.001 to 0.007)	.10	
**Specificity**					0.284
	Global region (reference: Temperate Northern)	Temperate Southern	–0.019 (–0.137 to 0.099)	.74	
	Global region (reference: Temperate Northern)	Tropical	0.143 (0.046 to 0.240)	.006	
**Timeliness**					0.430
	Log (total reports)	1 log	0.074 (–0.015 to 0.164)	.09	
	Official language (reference: not English)	English	–0.212 (–0.520 to 0.095)	.17	
	Latitude	1°	0.008 (0.003 to 0.014)	.006	

## Discussion

### Main Findings

To our knowledge, this study is the first to rigorously evaluate 2 EBS systems, HealthMap and EIOS, against a gold standard on a global scale, permitting the quantification of global variation in EBS performance and identification of country-specific factors determining this variation. High generalizability was guaranteed because 24 countries from 15 influenza transmission zones worldwide were included with multiple outbreak patterns of seasonal influenza.

We introduced the metric of timely sensitivity to assess the ability of each system to detect infectious disease outbreaks before or around the same time as traditional surveillance systems. Contrasting sensitivity with timely sensitivity highlights the discrepancy between the proportion of outbreaks that the systems provide information on (175/238, 73.5%) and the proportion of outbreaks where EBS could have led to early detection (22/238, 9.2%). These results demonstrate that monitoring report frequency in EBS may be insufficient for outbreak detection, and they suggest the need to reconsider assumptions about how EBS systems should be used to achieve timely outbreak detection across countries.

As we documented a significant variability in sensitivity, PPV, and timeliness of outbreak detection across countries, we analyzed several covariates as the potential drivers of this variability. We found that the number of reports gathered explained the performance variability of both systems, while a higher HDI improved HealthMap’s sensitivity and timeliness but decreased PPV. The most important predictor of EIOS performance was a country’s geographic location, with higher sensitivity, timeliness, and PPV, but reduced specificity in countries further away from the equator, indicating that EIOS is better in detecting clearly seasonal epidemic patterns. Surprisingly, EBS filter language was not found to be a determinant of between-country variation. Overall, the results suggest that both EBS systems had the best performance in high-income countries, although the systems failed to detect a considerable number of outbreaks in a timely manner in these countries. These results do not necessarily conflict with the findings that EBS does confer a timeliness advantage over traditional surveillance systems in the detection of Ebola or dengue fever [12-14].

The important influence of the number of gathered reports on system performance is likely due to measured and unmeasured factors. Unexpectedly, in our data set, there were no significant correlations between the total number of reports and a country’s HDI or PFI. Unmeasured factors that would explain this finding could be the country-specific news landscape, which news sources are included in news aggregators filtered by the EBS systems, and local internet availability and usage. With growing connectedness in low-income countries, the opportunity for the usage of social media arises. For example, during the Ebola epidemic in Western Africa in 2014/2015, monitoring of Twitter activity was retrospectively shown to produce earlier alerts than alerts by official bodies [14,35]. However, using social media as a data stream for disease surveillance raises additional challenges such as limited representativeness of the general public by social media users and a strong potential for misinformation [36,37].

Social media surveillance is an interesting way to complement current EBS [38,39], but from an evaluation perspective, it is imperative to first understand the performance of individual data streams before analyzing combined data sources. Since the number of gathered reports has an important influence on system performance, the usefulness of setting thresholds on report numbers for alerting EBS system users could be an interesting avenue to explore. For example, to contextualize information, alerts based on low reports numbers could be flagged and provided with a warning that EBS system information is likely unreliable because it is based on too little information.

### Comparison With Prior Work

Although there are many approaches to digital disease surveillance in general and digital influenza surveillance [40,41], in particular, we focused our evaluation on the utility of 2 EBS systems, which are based on digital news media. We focused on these systems as they are used routinely in public health practice internationally and within many countries. Given their regular use, it is important to develop a sound evidence base to guide their effective implementation and operation. In addition to EBS, many other approaches to digital disease surveillance have been proposed, including surveillance of web-based search queries, social media, or participatory online systems specifically for influenza. Although some of these approaches have gained attention, they are not widely used in public health practice [42]. One example is Google Flu Trends, a system developed by Google researchers using web-based search queries to nowcast regional influenza activity, which was found to correlate well with IBS systems and predict influenza-like illness incidence accurately [5]. However, Google Flu Trends was discontinued after it failed to detect the A/H1N1 pandemic and overestimated the 2012-13 influenza season [40,41,43]. Monitoring social media, mainly Twitter, has also shown potential for predicting disease outbreaks in multiple studies and correlates well with IBS data [44,45], but its use has been sporadic, often with a focus on large gatherings.

In one of the few published studies to quantify the performance of implemented EBS systems, Barboza et al [46] found that HealthMap detected H5N1 outbreaks worldwide on average 12 days before their gold standard with a detection rate of 43% and a PPV of 12% (compared to 75% and 66% in our study, respectively). Discrepancies may be due to differences in study design, as the authors of that paper took the first report of an H5N1 outbreak as the epidemic start point and used different gold standards. Interestingly, in the same study, the authors simulated a virtual system by aggregating data from 6 sources and assessed its performance [46]. This virtual combined system achieved a 93% detection rate of H5N1 outbreaks but only a 7% PPV as compared to a lower mean sensitivity (73%) but higher mean PPV (60%) of EIOS—the realization of a combined system—in our study. In another study, Barboza et al [18] also identified system type, filter language, outbreak region, and type of infectious disease as determinants of system performance but did not assess how the effect of these determinants varied by country.

### Limitations of This Study

Despite the use of influenza in other evaluations of EBS systems [46], there are limits to how well findings from such evaluations generalize to other infectious disease outbreaks due to the clear seasonality, high prevalence, and consequent potential lack of newsworthiness of influenza. Moreover, influenza shares important keywords for digital disease detection with other conditions of interest such as the common cold. Consequently, we expect our results may underestimate EBS performance because outbreaks of common infections such as influenza are likely more difficult to detect through EBS compared to less frequent and more newsworthy infectious diseases such as Ebola. Indeed, as EBS detection mechanisms might differ between communicable diseases more generally, we suggest separate performance evaluations of EBS systems for the detection of other types of diseases, using this study as a blueprint. For example, in the case of a hemorrhagic fever such as Ebola, EBS systems might give a greater lead in detection over traditional surveillance systems due to large reporting delays and poor data availability [13,14,47]. However, quantitative evaluation of EBS for diseases such as hemorrhagic fevers is hampered by the small number of outbreaks and the limited availability of gold standard data, particularly early in an outbreak. For these reasons and given the small number of EBS evaluation studies, we aimed to first advance the understanding of EBS performance by using a disease with sufficient report numbers to make valid statistical inferences. Traditional influenza surveillance through FluNet provides such a gold standard with high report numbers and almost global coverage. 

However, there were some limitations to FluNet for this evaluation. Most notably, epidemic intervals were not labelled explicitly in these data; therefore, we had to apply a statistical method to detect influenza outbreaks, allowing these intervals to labelled in the gold standard data. Although this approach appeared to work well for most countries, the performance was not good in Nigeria, Thailand, and Vietnam due to the low number of reported cases and the inherently more irregular influenza activity in these countries. Moreover, as FluNet influenza counts represent only people having sought health care, the total amount of influenza activity is underestimated, and differences in health care resources and surveillance activities may influence the number of specimens reported. A further complication is that some countries modify their testing and surveillance strategies over the course of an epidemic [48]. For instance, France reported influenza cases to FluNet only from the beginning of October to the beginning of May of each year.

Examples of other gold standard data used to evaluate EBS include the Centers for Disease Control and Prevention Influenza-like Illness Surveillance Network [5], WHO reports on H5N1 [46], and the Centers for Disease Control and Prevention Yellow Book [12]. All these data are dependent on health care–seeking behaviors or passive reporting from health care providers. Moreover, WHO and Yellow Book reports experience delays as they require official notification by a national authority. In fact, one could argue that a proper gold standard for influenza (or any other disease) does not exist, as all IBS systems are affected by delays, overreporting, and underreporting [49,50] and frequently capture only those cases seeking medical care [51]. The lack of a reliable gold standard for comparing the performance of EBS systems not only leads to differential results in the evaluation metrics for multiple systems but also creates problems when comparing the same system across diseases and regions. Given the challenges in identifying a suitable gold standard, we believe that the FluNet is a reasonable choice as it displays many characteristics of a good gold standard: (1) influenza cases are laboratory-confirmed, so FluNet is highly specific and has a high PPV for cases. These features are more important for determining the start and end of the epidemic than sensitivity (ie, absolute case counts), as laboratory-confirmed case counts accurately reflect the start and end points of epidemic periods, which is the information of interest in the gold standard in this case; (2) case count numbers are sufficiently high to make valid statistical inferences; (3) FluNet data cover a wide range of countries allowing measurement across many regions and seasons; and (4) our retrospective data extraction avoids the introduction of errors due to data delays and corrections. Moreover, the automated epidemic detection process we chose to apply likely differs from how EBS systems are used in practice. However, detection of outbreaks with BCP was a standardized way of looking at report frequency.

The variation of the performances of the systems could be explored but was limited. Since data were only available for 24 countries, regressions had to be performed with a small number of degrees of freedom and low numbers of countries per category in the categorical variables. Therefore, it was not possible to disentangle the effect of all variables in every situation.

Finally, the aggregation of daily EBS reports into weekly counts was an important limitation affecting our capacity to ascertain timely sensitivity, but it was necessary to guarantee comparability with the gold standard data. Similarly, as the EBS data were aggregated per country, they did not capture any regional diversity within countries. This is especially problematic for noncontiguous landmasses and large countries spanning diverse climatic regions such as Brazil and China, with different epidemic properties of influenza [48], but again necessary to guarantee comparability.

### Conclusion

As the SARS-CoV-2 pandemic has made clear, infectious diseases will continue to be major risks for global health security. The results from this study can help to guide development toward better EBS to prevent future large outbreaks. As demonstrated by the poor timely outbreak detection in this study, advances in the use and evaluation of EBS are needed.

Our analysis documented considerable performance variations across settings. Depending on what exists as routine public health surveillance, related infrastructure, and media landscape, there are situations where EBS can currently be useful, especially in high-income countries for seasonal influenza epidemic surveillance. Efforts need to be made to better understand the determinants of outbreak detection through EBS, particularly in low- and middle-income countries, as current EBS systems were found to be a disadvantage in tropical regions and regions with lower HDI. The inequalities created by biases in EBS, such as low media and internet coverage and low newsworthiness of tropical diseases, should be explored in future research, and the needs of resource-poor settings should be met through further development of EBS.

## Appendix

Multimedia Appendix 1 Supplementary methods.

Multimedia Appendix 2 Mean annual report numbers relating to influenza outbreaks in HealthMap (from January 2013 to July 2019) and Epidemic Intelligence from Open Sources (from November 2017 to December 2019). EIOS: Epidemic Intelligence from Open Sources.

Multimedia Appendix 3 Evaluation metrics of HealthMap (from January 2013 to July 2019) and Epidemic Intelligence from Open Sources (from November 2017 to December 2019) for the detection of influenza outbreaks in the United States and India. EIOS: Epidemic Intelligence from Open Sources.

## Abbreviations

BCP: Bayesian change point
EBS: event-based surveillance
EIOS: Epidemic Intelligence from Open Sources
GISRS: global influenza surveillance and response system
HDI: human development index
IBS: indicator-based surveillance
PFI: Press Freedom Index
PPV: positive predictive value
WHO: World Health Organization
