# Supplementary material for: Global Variations in Event-Based Surveillance for Disease Outbreak Detection: Time Series Analysis
Source: JMIR Public Health Surveill. 2022 Oct 31;8(10):e36211. doi: 10.2196/36211 (PMC9664335; doi:10.2196/36211)
Supplement: Multimedia Appendix 1 [file publichealth_v8i10e36211_app1.docx]

## Supplementary Methods

BCP analysis estimates the posterior probability of each observation *i* being a change point (*p_i_*), as well as the posterior mean and variance at each *i*. The parameters and positions of change points are estimated with a Markov Chain Monte Carlo (MCMC) approximation, which is implemented in the *bcp* package. For each *i*, *p_i_* is calculated as:

$$\frac{p_{i}}{1-p_{i}}= \frac{P\left( U_{i}=1 \right|X, U_{j}, j\neq i)}{P\left( U_{i}=0 \right|X, U_{j}, j\neq i)}= \frac{\left[ \int_{0}^{p_{0}} p^{b}\left( 1-p \right)^{n-b-1}dp \right]\left[ \int_{0}^{w_{0}} \frac{w^{\frac{b}{2}}}{{{(W}_{1}+B_{1}w)}^{\frac{n-1}{2}}}dw \right]}{\left[ \int_{0}^{p_{0}} p^{b-1}\left( 1-p \right)^{n-b}dp \right]\left[ \int_{0}^{w_{0}} \frac{w^{\frac{b-1}{2}}}{{{(W}_{0}+B_{o}w)}^{\frac{n-1}{2}}}dw \right]}$$

where *U_i_* = 1 indicates a change point at position i, and *U_i_* = 0 indicates no change point. p. *p_0_* and *w_0_* are the priors on the probability of a change point at each position and the signal-to-noise ratio, respectively. *b* is the number of blocks found if *U_i_* = 0. *W_1_* and *W_0_* are the within-block sum of squares when *U_i_* = 1 and *U_i_* = 0, respectively. Likewise, *B_1_* and *B_0_* are the between-block sum of squares. w=σ^2^/(σ_0_^2^+σ^2^), where σ_0_^2^ is the overall variance and σ^2^ is the block variance, so *w* represents the signal-to-noise ratio. Intuitively, *p_i_* is larger when *W_1_* small and *B_1_* is large.

At each step of the Markov chain, a value for *U_i_* is drawn from the conditional distribution of *U_i_*, given the data and the current segmentation of observations into blocks. Based on these values, *p_i_* is calculated with the above equation. After every MCMC iteration, *p_i_* and the posterior mean of each block are updated. The posterior mean for every block is calculated with $\hat{\mu}_{ij}=\left( 1-w \right)\bar{X}_{ij}+w\mu_{0}$where $\bar{X}_{ij}$is the mean of the observations in the block *b_ij_*.

As multiple change points were flagged during outbreaks, the following criteria were established to determine the start and end points of influenza outbreaks: Epidemic ‘start’ points were located on rising count curves, no ‘start’ had been flagged during previous 15 weeks, and the ‘start’ point was the transition point from $p_{i}$ < 0.5 to $p_{i}$ ≥ 0.5. Epidemic ‘end’ points were on decreasing count curves, no ‘end’ was flagged during following 15 weeks, and an ‘end’ point was at the transition from $p_{i}$ ≥ 0.5 to $p_{i}$ < 0.5. Based on start and end points of epidemics, the datasets were divided into ‘epidemic’ and ‘nonepidemic’ periods. The 15-week period of no start points before a start point and after an end was chosen so that the algorithm would not flag epidemic ‘starts’ or ‘ends’ during an ongoing outbreak. Choosing 15 weeks as the interval between two ‘starts’ will therefore miss an epidemic only if the non-epidemic period between two outbreaks is shorter than two weeks. When detecting spikes, the algorithm usually flagged an epidemic start point, but often no end point. In order to allow detection of spikes as single outbreak weeks, an ‘end’ was inserted after every ‘start’ that was not followed by an ‘end’ within a period of 30 weeks.
