# Supplementary figures and images for: Global Variations in Event-Based Surveillance for Disease Outbreak Detection: Time Series Analysis
Source: JMIR Public Health Surveill. 2022 Oct 31;8(10):e36211. doi: 10.2196/36211 (PMC9664335; doi:10.2196/36211)

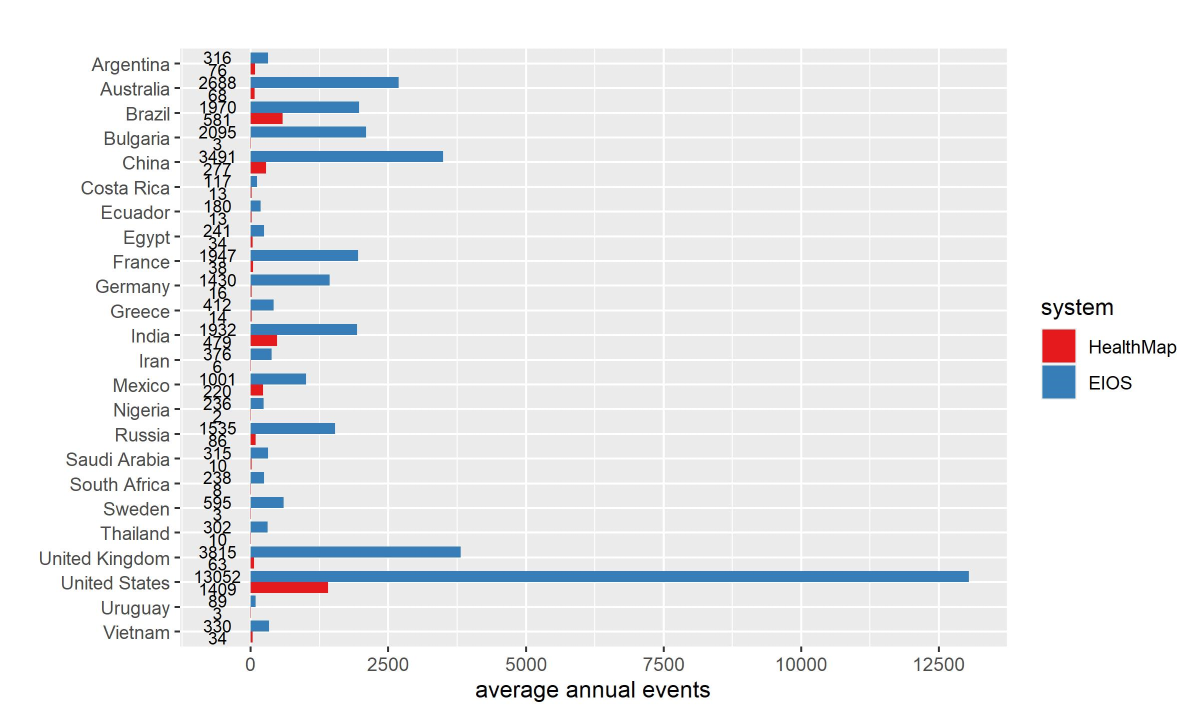

Supplement: Multimedia Appendix 2 [file publichealth_v8i10e36211_app2.png]

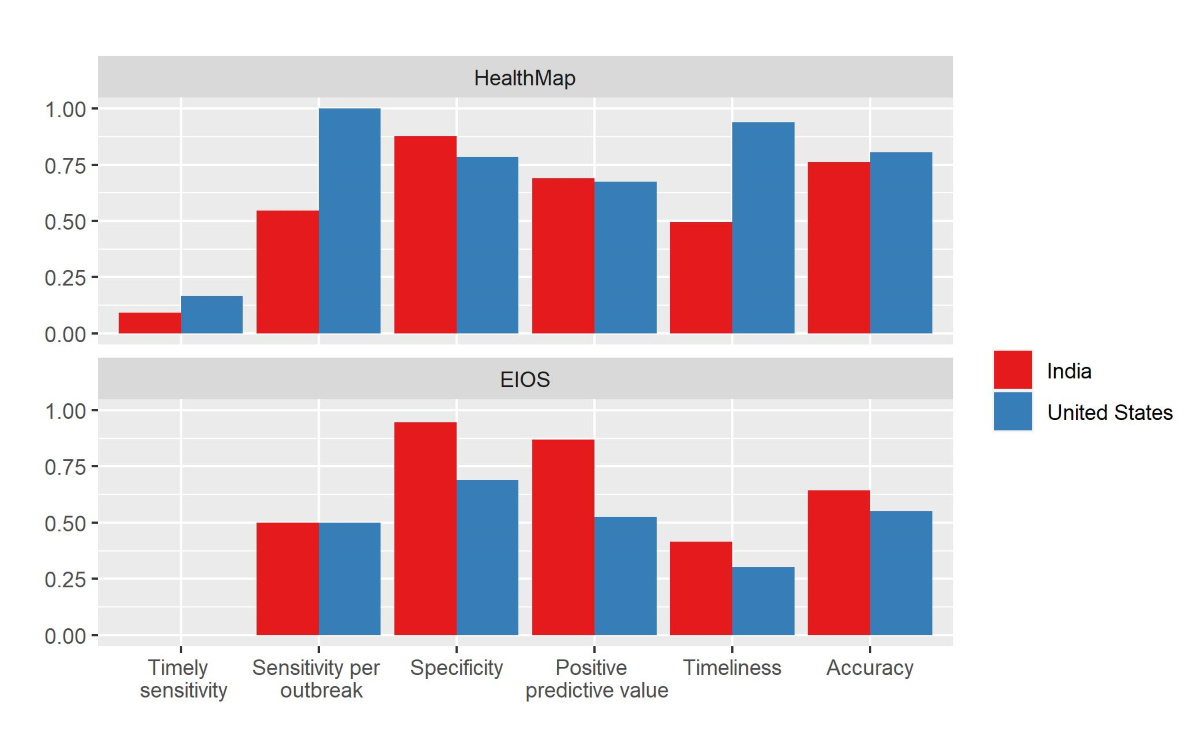

Supplement: Multimedia Appendix 3 [file publichealth_v8i10e36211_app3.png]
